# Retinal degeneration-3 protein promotes photoreceptor survival by suppressing activation of guanylyl cyclase rather than accelerating GMP recycling

**DOI:** 10.1016/j.jbc.2021.100362

**Published:** 2021-02-02

**Authors:** Alexander M. Dizhoor, Elena V. Olshevskaya, Igor V. Peshenko

**Affiliations:** Pennsylvania College of Optometry, Salus University, Elkins Park, Pennsylvania, USA

**Keywords:** calcium-binding proteins, cyclic GMP, GCAP, GMP, guanylate cyclase (guanylyl cyclase), retinal degeneration, congenital blindness, photoreceptor, RD3, RetGC, signal transduction, vision, ERG, electroretinography, GCAP, guanylyl cyclase activating protein, LCA12, Leber’s congenital amaurosis-12, PDE6, cGMP phosphodiesterase 6, PEI, polyethylenimine, RD3, retinal degeneration 3 protein, RetGC, retinal membrane guanylyl cyclase

## Abstract

Retinal degeneration-3 protein (RD3) deficiency causes photoreceptor dysfunction and rapid degeneration in the *rd3* mouse strain and in human Leber’s congenital amaurosis, a congenital retinal dystrophy that results in early vision loss*.* However, the mechanisms responsible for photoreceptor death remain unclear. Here, we tested two hypothesized biochemical events that may underlie photoreceptor death: (i) the failure to prevent aberrant activation of retinal guanylyl cyclase (RetGC) by calcium-sensor proteins (GCAPs) *versus* (ii) the reduction of GMP phosphorylation rate, preventing its recycling to GDP/GTP. We found that GMP converts to GDP/GTP in the photoreceptor fraction of the retina ∼24-fold faster in WT mice and ∼400-fold faster in *rd3* mice than GTP conversion to cGMP by RetGC. Adding purified RD3 to the retinal extracts inhibited RetGC 4-fold but did not affect GMP phosphorylation in wildtype or *rd3* retinas. RD3-deficient photoreceptors rapidly degenerated in *rd3* mice that were reared in constant darkness to prevent light-activated GTP consumption *via* RetGC and phosphodiesterase 6. In contrast, rd3 degeneration was alleviated by deletion of GCAPs. After 2.5 months, only ∼40% of photoreceptors remained in *rd3*/*rd3* retinas. Deletion of GCAP1 or GCAP2 alone preserved 68% and 57% of photoreceptors, respectively, whereas deletion of GCAP1 and GCAP2 together preserved 86%. Taken together, our *in vitro* and *in vivo* results support the hypothesis that RD3 prevents photoreceptor death primarily by suppressing activation of RetGC by both GCAP1 and GCAP2 but do not support the hypothesis that RD3 plays a significant role in GMP recycling.

RD3 protein (1) contributes to the physiological function of rods and cones by promoting accumulation of retinal membrane guanylyl cyclase (RetGC) in the photoreceptor outer segment (2, 3, 4). RetGC produces cGMP, the second messenger of phototransduction, which opens cGMP-gated CNG channels in the outer segment and lets the inward Na^+^/Ca^2+^ current partially depolarize photoreceptors in the dark (reviewed in (5, 6, 7)). Light-activated phosphodiesterase 6 (PDE6) hydrolyzes cGMP to 5’GMP and shuts off the cation influx *via* CNG channels, thus hyperpolarizing photoreceptors in the light. Consequently, in response to the decrease of Ca^2+^ influx, RetGC accelerated by Mg^2+^-liganded calcium-sensor proteins (GCAPs) (reviewed in references (5, 8)), accelerate photoreceptor recovery, and light adaptation by reopening CNG channels (9, 10, 11, 12, 13), also reviewed in (13, 14). RD3 deficiency reduces RetGC content in the photoreceptor outer segment and suppresses retinal photoresponses (1, 15), making rods and cones dysfunctional from birth in recessive human Leber’s congenital amaurosis-12 (LCA12) (1, 16) and *rd3* mouse strain (1). RD3 not only enables the normal function of rods and cones but also is essential for their survival—in addition to the reduction of rod and cone responses, the lack of RD3 causes rapid progressive degeneration of the photoreceptors (1).

A previous study demonstrated that in homozygous *rd3/rd3* mice (genotype synonymous with *Rd3*^*-/-*^), where a nonsense mutation similar to LCA12 in humans truncates RD3 (1), photoreceptors degenerate much more rapidly than in mice completely lacking RetGC (17). Therefore, the reduction of RetGC activity per se does not constitute the principal cause of apoptosis in RD3-deficient rods and cones. In previous studies, we established that RD3 strongly inhibits RetGC activity (17, 18, 19). When the residual RetGC in *rd3* photoreceptors cannot be stimulated by GCAPs, this drastically slows their degeneration, despite further reduction in their cGMP production rate (17, 19). These observations gave rise to the hypothesis that RD3 protects photoreceptors against rapid degeneration primarily by blocking aberrant activation of RetGC by GCAPs in the inner segment (17, 18, 19). In this study, we further evaluated this hypothesis by comparing the effect of deleting GCAP1 and the effect of deleting GCAP2 on *rd3* photoreceptor degeneration.

We also tested an alternative hypothesis recently proposed by Wimberg *et al.* (20). This hypothesis suggests that the RD3-deficient photoreceptors die because they are unable to stimulate RD3-dependent phosphorylation of GMP. The decay of GTP to GMP in photoreceptors accelerates in the light, when RetGC converts GTP to cGMP and PDE6 subsequently hydrolyses cGMP to 5’GMP (reviewed in (2, 3, 4)). According to the Wimberg *et al*. (20) hypothesis, RD3 is required to stimulate guanylate kinase in the inner segment to recycle GMP back to the GDP pool. Consequently, in the absence of RD3, photoreceptors cannot phosphorylate GMP and fail to replenish GTP consumed by RetGC (20).

In our study, we compared the rates of GMP phosphorylation and GTP consumption in photoreceptors of the normal and RD3-deficient mice and tested how RD3 affected the rate of GMP recycling to the GDP/GTP pool. The results of this study support the hypothesis that the protective role of RD3 against photoreceptor degeneration is to counteract stimulation of RetGC by both GCAP1 and GCAP2. In contrast, the results also rule out the possibility that RD3 deficiency causes photoreceptor degeneration by making them unable to recycle GMP produced *via* the light-activated RetGC/PDE6 pathway.

## Results

### Both GCAP1 and GCAP2 contribute to the degeneration of RD3-deficient photoreceptors

**Figure 1:**
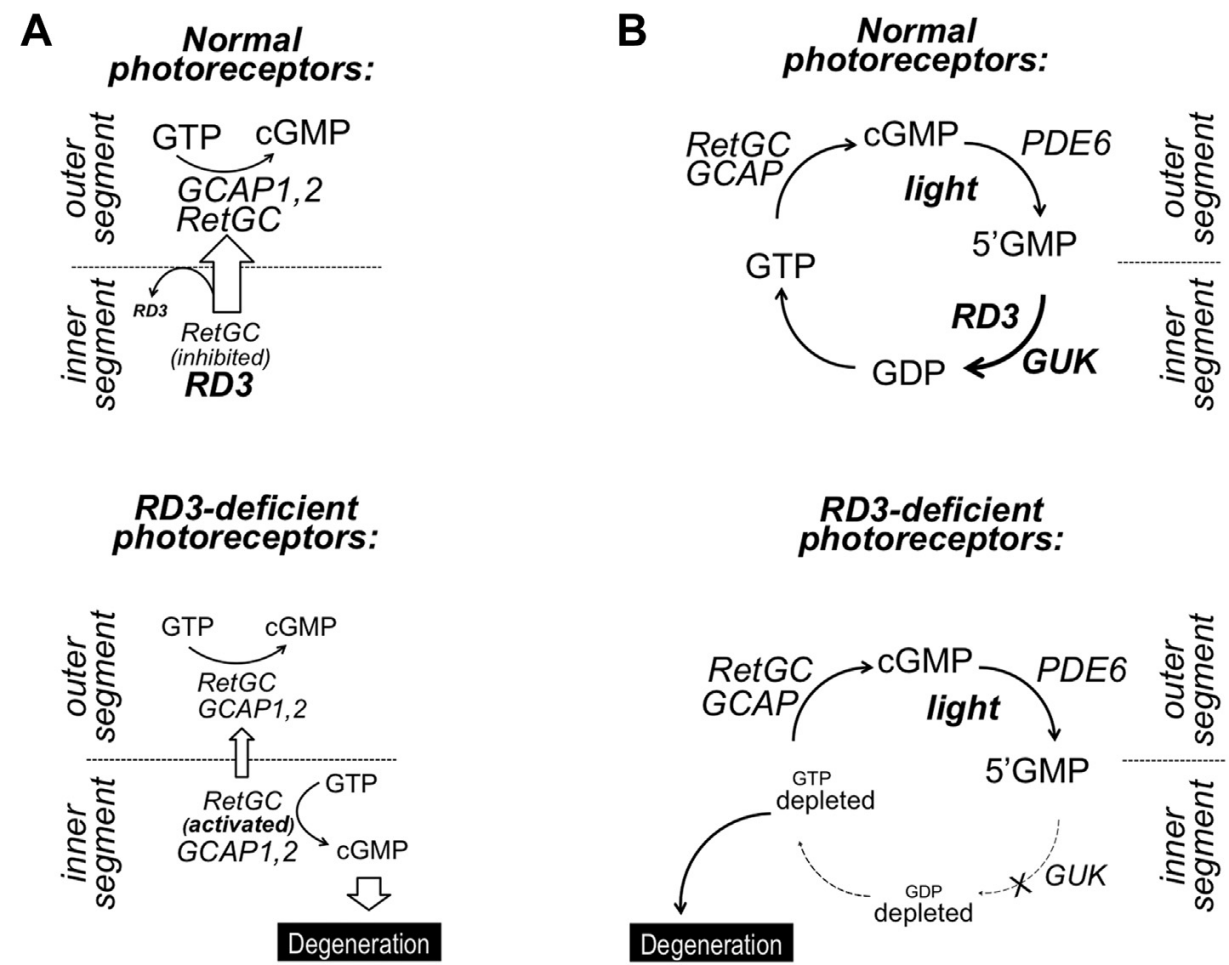
**Two hypothetical roles of RD3 in preventing photoreceptor degeneration.***A*, Hypothesis 1: RD3 is required to suppress aberrant activation of RetGC cyclase by GCAPs (17, 18, 19). In normal photoreceptors, the membrane guanylyl cyclase (RetGC) is delivered with the help of RD3 to the outer segment, where it produces cGMP for phototransduction. While in the inner segment, the RetGC is suppressed by RD3 blocking the cyclase activation by GCAPs. In RD3-deficient photoreceptors, RetGC content in the outer segment is strongly reduced, but the cyclase remaining in the inner segment becomes unprotected against activation by GCAPs, and this triggers the apoptotic process. *B*, Hypothesis 2: RD3 accelerates GMP phosphorylation by guanylate kinase (20). In the light, RetGC rapidly converts GTP to cGMP, and then PDE6 converts cGMP to 5’GMP. RD3 stimulates guanylate kinase activity to convert GMP to GDP and then back to GTP. The RD3-deficient photoreceptors fail to phosphorylate GMP; therefore, the RetGC/PDE6 pathway depletes the GDP/GTP pool and thus causes photoreceptor degeneration (20). GCAP, guanylyl cyclase activating protein; RD3, retinal degeneration 3 protein; RetGC, retinal membrane guanylyl cyclase; PDE6, cGMP phosphodiesterase 6.

**Figure 2:**
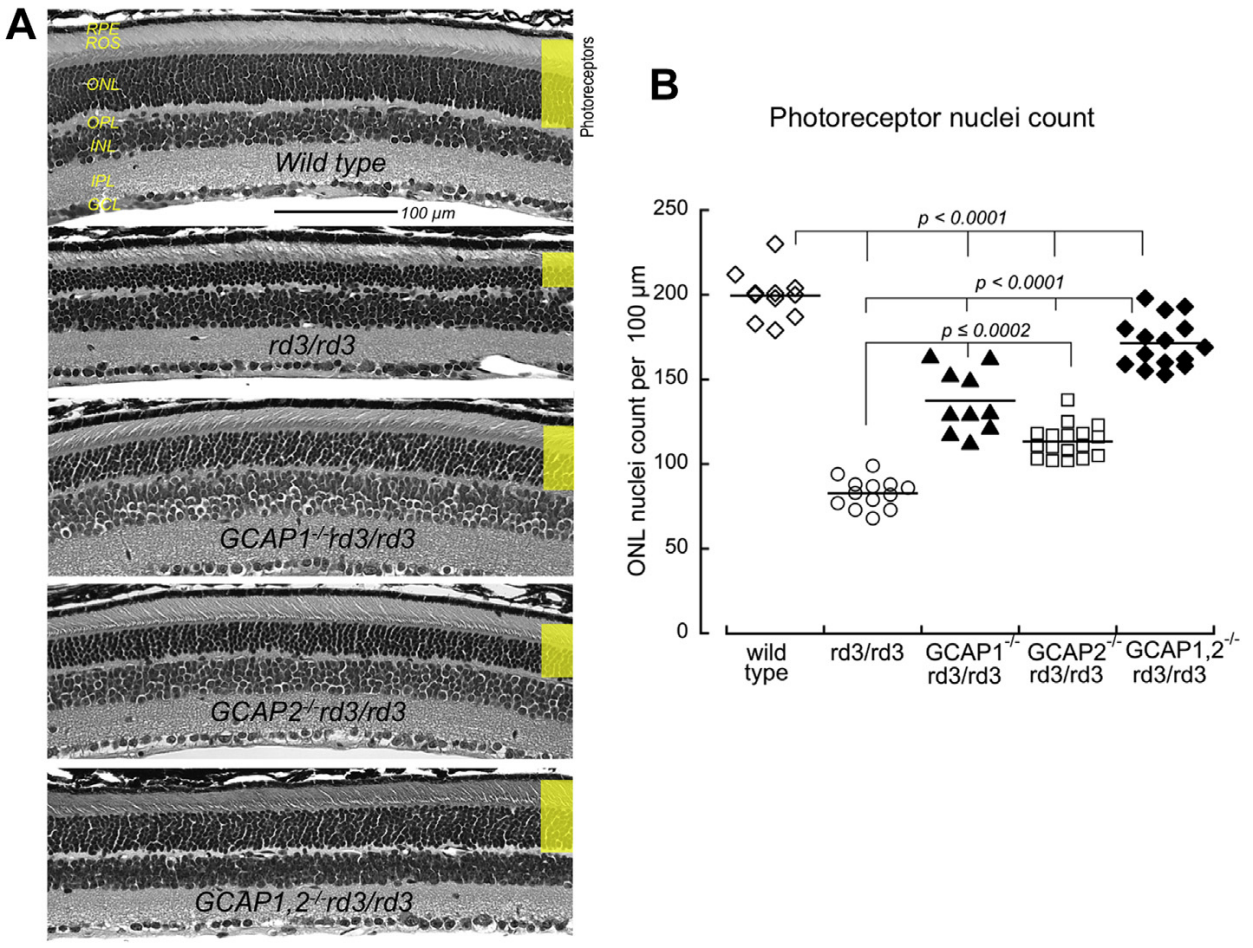
**Deletion of calcium-sensor activators of guanylyl cyclase rescues *rd3/rd3* photoreceptors.***A*, representative retinal morphology at 2.5 months of age in wildtype, *rd3/rd3*, *GCAP1*^*-/-*^*rd3/rd3*, *GCAP2*^*-/-*^*rd3/rd3*, and *GCAP1,2*^*−/−*^*rd3/rd3*; hereafter, the retinal histological layers are referred to as *RPE* (retinal pigment epithelium), *ROS* (rod outer segments), *ONL* (outer nuclear layer), *OPL* (outer plexiform layer), *INL* (inner nuclear layer), *IPL* (inner plexiform layer), and *GCL* (ganglion cell layer). The photoreceptor layer thickness between the retinal pigment epithelium and outer plexiform layer is highlighted in *yellow*. *B,* photoreceptor nuclei count in the outer nuclear layer at 2.5 months in wildtype (◇), *rd3/rd3* (○), *GCAP1*^*-/-*^*rd3/rd3* (▲), *GCAP2*^*-/-*^*rd3/rd3* (□), and *GCAP1,2*^*−/−*^*rd3/rd3* (♦); each data point is from a different mouse. The differences were tested using ANOVA (F = 158, *p* < 0.0001); the *p*-values shown in the graph are from the Tukey’s HSD *post hoc* comparison (hereafter, confidence level 99%, alpha = 0.01). GCAP, guanylyl cyclase activating protein.

Figure 1*A* illustrates the hypothesis that an aberrant GCAP-stimulated RetGC activity in the inner segment triggers *rd3* degeneration (17, 18, 19). GCAP1 and GCAP2 are the two ubiquitous forms of GCAPs in vertebrate species (21, 22, 23), and the only two isoforms present in rodents (10). Simultaneous disruption of the two adjacent genes, *Guca1a* and *Guca1b* (10), respectively, coding for GCAP1 and GCAP2 slowed *rd3* photoreceptor degeneration in *GCAP1,2*^*−/−*^*rd3/rd3* mouse retinas (19, 24). Plana-Bonamaisó *et al.* (24) proposed that the *rd3* apoptosis is triggered primarily by GCAP2 retained in the inner segment. Therefore, we bred the *rd3* mice in either *GCAP1*^*-/-*^ (25) or *GCAP2*^*-/-*^ (26) backgrounds and compared the extent of the photoreceptor degeneration in the resultant *GCAP1*^*-/-*^*rd3/rd3* and *GCAP2*^*-/-*^*rd3/rd3* genotypes with that in the *rd3/rd3* or *GCAP1,2*^*−/−*^*rd3/rd3* genotypes (Fig. 2). RD3-deficient photoreceptors in a C57Bl6 strain background completely degenerate after 6 months of age (17, 19), with the rate of photoreceptor loss being the highest within the first 4 months. We compared different genotypes at the age of 2.5 months, the time point when any potential effects on acceleration or deceleration of the degeneration would be most noticeable. At 2.5 months, the average photoreceptor nuclei count per 100-μm length of the retina in *rd3/rd3* mice was reduced to 42% of the normal count (83 ± 9, *n* = 13, *versus* 200 ± 14, *n* = 11, *p* < 0.0001). The removal of GCAP1 or GCAP2 alone increased the average *rd3/rd3* photoreceptor nuclei count to 68% and 57%, respectively (137 ± 19, *n* = 10, and 113 ± 10, *n* = 17, *p* = 0.0002), whereas deletion of both GCAPs most efficiently offset the degeneration, preserving 86% of RD3-deficient photoreceptors (171 ± 14, *n* = 15, *p* = 0.0001).

### Comparison of guanylate kinase and guanylyl cyclase activities in normal and rd3/rd3 mouse retinas

We further compared GMP phosphorylation and cGMP production rates in the normal and *rd3* retinas to test Wimberg *et al*.’s hypothesis (20) (Fig. 1*B*). We emphasize that we estimated only the depletion of GTP to GMP *via* RetGC1/PDE6 activity, hypothetically counteracted by RD3, not the depletion of the GTP *via* GTPase activities, such as that of transducin (5, 6, 7), because the latter would not deplete the GDP pool in the model proposed by Wimberg *et al*. (20) (Fig. 1*B*).

**Figure 3:**
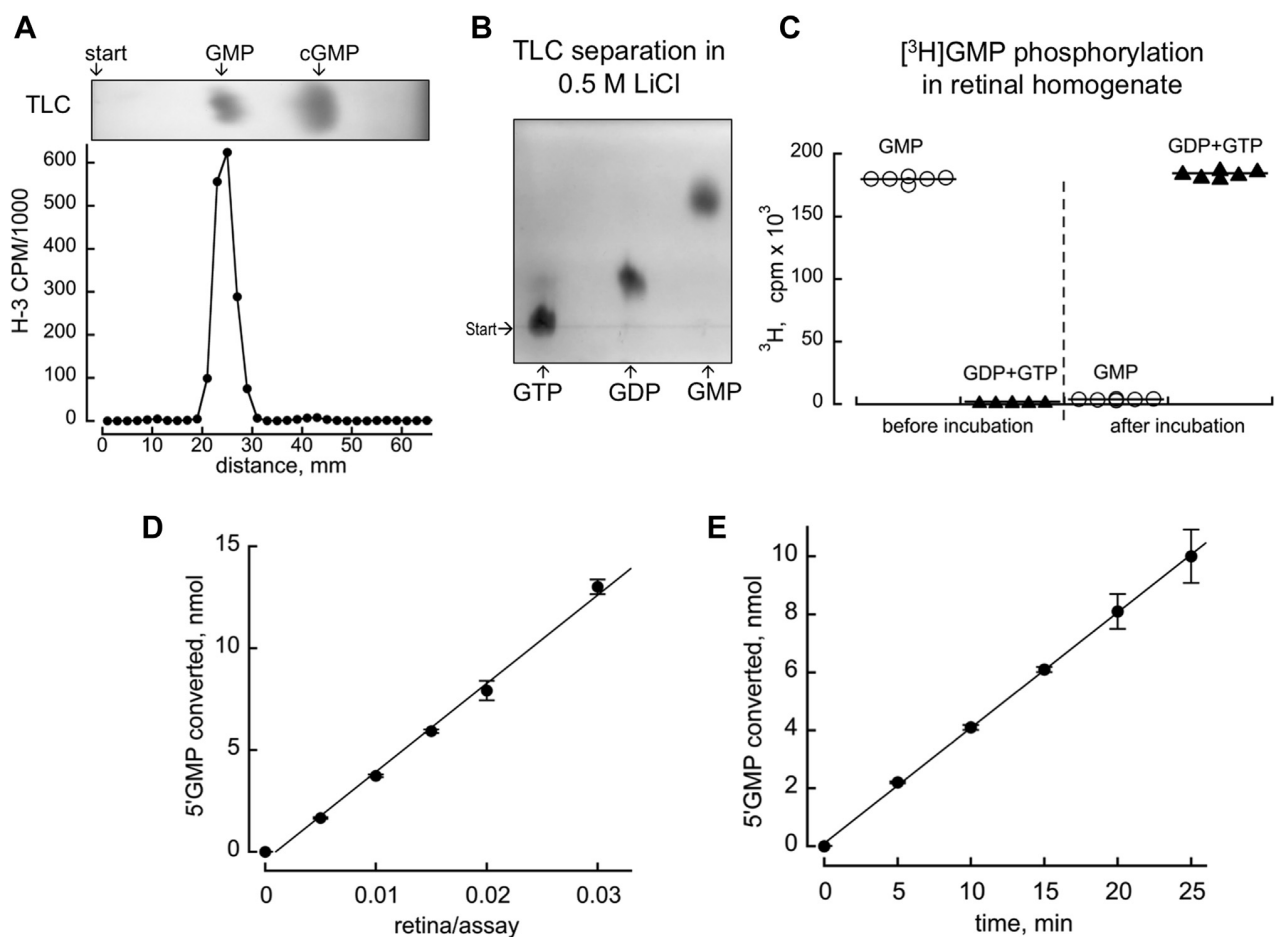
**GMP phosphorylation assay.** The GMP phosphorylation was assayed as described in the Experimental Procedures. *A,* The [^3^H]GMP was produced by converting [^3^H]cGMP by PDE6, and the resultant preparation (analyzed by TLC on PEI cellulose plates) was used as the substrate in the GMP phosphorylation assay. *B,* Separation of GTP, GDP, and GMP using TLC. *C,* Incubation of [^3^H]GMP with the retinal extract converts it to the phosphorylated forms, GDP + GTP. *D, E,* GMP phosphorylation yield was directly proportional to the amount of retinal homogenate (*D*) and was linear with time (*E*) in the conditions of the assay; mean ± SD, *n* = 3 technical repeats. PDE6, cGMP phosphodiesterase 6; PEI, polyethylenimine.

To avoid potential nonspecific effects of RD3 on a complex enzymatically coupled spectrophotometric assay used in study by Wimberg *et al.* (20), we directly monitored [^3^H]GMP phosphorylation, combining its conversion to [^3^H]GDP by guanylate kinase and subsequently to [^3^H]GTP by nucleoside diphosphokinase, highly active in photoreceptors (27), with a possible contribution from succinyl-CoA synthetase of the Krebs cycle in the mitochondrial matrix (28). The method utilized separation of the [^3^H]GMP substrate (Fig. 3*A*) from its phosphorylated products, [^3^H]GDP and [^3^H]GTP, using TLC on polyethyleneimine (PEI) cellulose (Fig. 3*B*). Incubation with the retinal extract resulted in a complete conversion of [^3^H]GMP to the phosphorylated species (Fig. 3*C*), indicating a lack of interference from any reversible reactions potentially capable of affecting the analysis. The concentrations of GMP and ATP in the assay (1 mM and 2 mM, respectively) by far exceeded the respective 14 μM k_m,GMP_ and 0.4 mM k_m,ATP_ values for the retinal guanylate kinase reported by Hall and Kuhn (29). [^3^H]GMP conversion to [^3^H]GDP/[^3^H]GTP in the conditions of the assay was directly proportional to the concentration of the retinal extracts and remained linear throughout the incubation period (Fig. 3, *D* and *E*).

**Figure 4:**
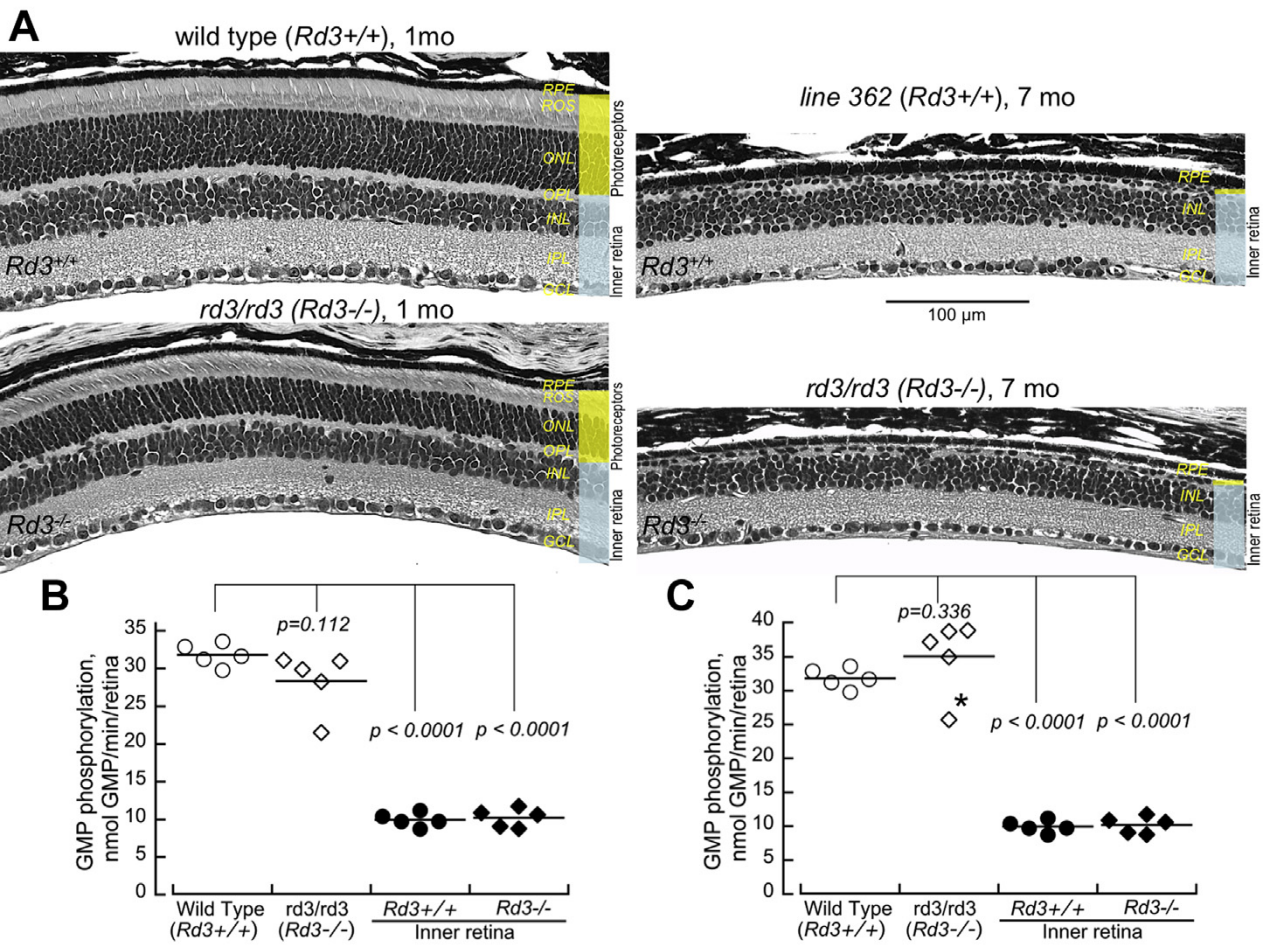
**GMP phosphorylation rate in RD3-deficient photoreceptors is similar to that in normal photoreceptors.***A,* The representative morphology of the retina: wildtype (*Rd3*^*+/+*^) at 1 month of age, *rd3/rd3* (*Rd3*^*-/-*^) at 1 month of age, line 362 (30, 31) (*Rd3*^*+/+*^) at 7 months, and *rd3/rd3* (*Rd3*^*-/-*^) at 7 months. Note the reduction of the photoreceptor-layer thickness in *rd3/rd3* and the complete lack of photoreceptors in the aged line 362 and *rd3/rd3*. The photoreceptor layer is marked *yellow,* and the inner retina layers are marked *blue*. *B* and *C,* GMP phosphorylation in whole-retina homogenates from wildtype (*Rd3*^*+/+*^) (○), *rd3/rd3* mice (*Rd3*^*-/-*^) (◇), in the inner *Rd3*^*+/+*^ retina from the aged 7 months line 362 mice (●), and the inner *Rd3*^*-/-*^ retina from the aged *rd3/rd3* (♦) mice; not corrected for the reduction of photoreceptor count (*B*) and corrected for the ∼29% loss of photoreceptor count in *rd3/rd3* retinas at 1 month of age (*C*); the *p*-values in the graph are from the ANOVA (F = 107, *p* < 0.0001)/Tukey’s HSD *post hoc* test. The activities in the inner *Rd3*^*+/+*^retina (line 362) or the inner *Rd3*^*-/-*^ (aged 7 months *rd3/rd3* retinas) were subtracted from the respective total activities in the wildtype and the young *rd3/rd3* retinas to assess the GMP phosphorylating activity that belonged to the layer of photoreceptors (summarized in Table 1). The sample of the *rd3/rd3* retinas marked with an *asterisk* was subsequently used to test the effect of the addition of purified recombinant RD3 to the assay (see Fig. 5). See the Experimental Procedures section for other details. RD3, retinal degeneration 3 protein.

In WT mouse retinas that retained normal complement of photoreceptors (Fig. 4*A*), GMP was phosphorylated at the rate of 32 nmol/min/retina (Fig. 4*B*, Table 1). However, the photoreceptor layer comprises only approximately half of the retinal tissue mass (Fig. 4*A*, highlighted in *yellow*). Therefore, to evaluate the fraction of GMP-phosphorylating activity in the photoreceptor layer, we compared the WT (*Rd3*^*+*/+^) retinas with the *Rd3*^*+*/+^ retinas from CORD6 transgenic mouse line 362 (30, 31), where photoreceptors completely degenerate around 6 months of age. To isolate the activity in the inner retina, we used 7-month-old retinas that are devoid of the photoreceptor layer but retain a visibly intact histological layer of the inner retinal neurons (marked *blue* in Fig. 4*A*). In the latter case, GMP was phosphorylated at the rate ∼10 nmol/min/retina. After subtracting the activity associated with the inner retina, the GMP phosphorylation rate corresponding to the photoreceptor layer equaled 24.7 nmol GMP/min/retina, comprising nearly 70% of the activity in the whole retina. We also used retinas from *rd3/rd3* mice aged 7 months, lacking the photoreceptor layer at that age (Fig. 4*A*), to determine the fraction of guanylate kinase activity in *Rd3*^*-/-*^ inner retina (Table 1). It was virtually indistinguishable from the *Rd3*^*+/+*^ line 362 mice (*p* > 0.999). In young *rd3/rd3* mouse retinas retaining ∼71% of the photoreceptors (Fig. 4*A*), the GMP to GDP/GTP conversion rate was ∼28 nmol GMP/min/retina, not significantly different from that in WT retinas (ANOVA/Tukey’s HSD, *p* = 0.11). After subtracting the activity in the inner retina, the average *rd3/rd3* photoreceptor-specific activity comprised ∼18 nmol GMP/min/retina (Table 1). When further corrected for the reduction of photoreceptor nuclei count, the photoreceptor-specific activity in *rd3* photoreceptors was estimated as ∼25 nmol GMP/min/retina and the total activity in the retina as ∼35 nmol GMP/min/retina; neither of these values was significantly different from those in WT retinas (Fig. 4*C*, Table 1).

**Table 1:** Guanylyl cyclase *versus* guanylate kinase activities in mouse retinal tissue

Tissue	Enzymatic activity
	RetGC (nmol cGMP/min/retina, mean ± SD):
WT (*Rd3*^*+/+*^) retina^a^	0.9 ± 0.2, n = 20
*rd3/rd3 (Rd3*^*-/*-^) retina^a^^,^^b^	0.06 ± 0.02, n = 9 (*p* < 0.0001)
	GMP phosphorylation (nmol GMP/min/retina, mean ± SD):
WT (*Rd3*^*+/+*^), whole retina	31.8 ± 0.15, n = 5
*Rd3*^*+/+*^, inner retina	10 ± 0.91, n = 5 (*p* < 0.0001)
WT, photoreceptor layer^c^	21.9 ± 1.74, n = 5
*rd3/rd3 (Rd3*^*-/*-^), whole retina	28.4 ± 4, n = 5 (*p* = 0.11)
*rd3/rd3 (Rd3*^*-/*-^), inner retina	10.2 ± 1.25 (*p* < 0.0001)
*rd3/rd3,* photoreceptor layer, not corrected for degeneration^c^	18.14 ± 4.18, n = 5 (*p* = 0.37)
*rd3/rd3,* photoreceptor layer, corrected for reduction of photoreceptor count^b^^,^^d^	24.9 ± 5.72 (*p* = 0.52)
*rd3/rd3*, whole retina, corrected for reduction of photoreceptor count^b^^,^^d^	35.1 ± 4.36 (*p* = 0.336)
	(GMP phosphorylation rate)/(RetGC rate)^e^
WT, whole retina	35.4 ± 8
WT, photoreceptor layer	24.3 ± 6
*rd3/rd3*, whole retina, not corrected for degeneration	473 ± 171 (*p* = 0.0046)
*rd3/rd3*, photoreceptor layer, not corrected for degeneration	302 ± 122 (*p* = 0.007)
*rd3/rd3*, photoreceptor layer, corrected for degeneration	415 ± 168 (*p* = 0.0065)
*rd3/rd3*, whole retina, corrected for degeneration	585 ± 208 (*p* = 0.0041)

RetGC, retinal membrane guanylyl cyclase.

Mouse retinas were extracted at 3.5 weeks of age and assayed as described in Figure 4. RetGC and guanylate kinase activities were assayed as described in Experimental procedures; *p*—statistical significance of the differences from the WT from ANOVA/Tukey’s HSD *post hoc,* unless indicated otherwise.

a The entire RetGC activity is present in photoreceptor layer and is undetectable in the inner retina (27, 38).

b Corrected for ∼28% reduction in photoreceptor count.

c The activity in the inner retina subtracted from the activity in whole retina of the respective genotype; (μ_1_ − μ_2_) ± $\sqrt{\text{σ}1^{2}+\text{σ}{2^{2}}^{\;}}$, where μ_1_ and μ_2_ are average activities in whole retina and the inner retina, and σ1 and σ2–their respective standard deviations. Propagation of error algorithms hereafter are from an open-access on-line resource Chemistry LibreTexts, https://chem.libretexts.org/@go/page/353

d The activity in photoreceptors plus in the inner retina; (μ_1_ + μ_2_) ± $\sqrt{\text{σ}1^{2}+\text{σ}{2^{2}}^{\;}}$; where μ_1_ the activity in *rd3* photoreceptors, μ_2_ is the average activity in the *Rd3*^*+/+*^ inner retina (aged line 362) or *Rd3*^-/-^ inner retina (aged *rd3/rd3* mice), σ1 and σ2–their respective standard deviations.

e Mean average (μ) = μ_ph_/μ_GC_; SD = $\text{μ }\times \;\sqrt{{\left(\sigma_{\text{ph}}/\mu_{\text{ph}}\right)}^{2}+{\left(\sigma_{\text{GC}}/\mu_{\text{GC}}\right)}^{2}}$; where μ_ph_ is the rate of GMP phosphorylation, μ_GC_ is the rate of GTP depletion *via* RetGC, and σ_ph_ and σ_GC_–the respective standard deviations; the *p-*values are from Student’s *t* test.

### RD3 inhibits RetGC activity but does not affect GMP phosphorylation rate in the retina

**Figure 5:**
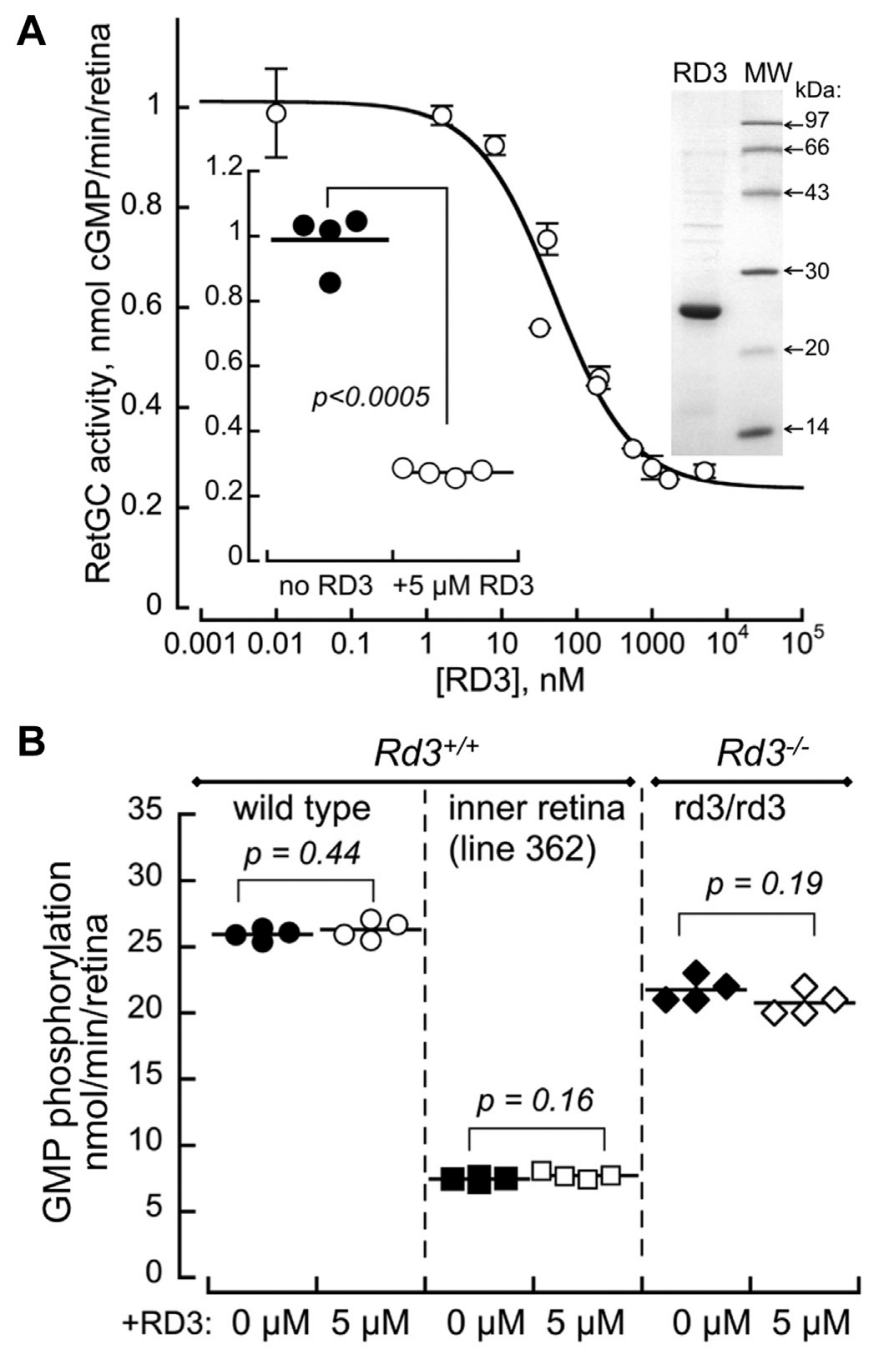
**RD3 strongly inhibits RetGC activity in the retina but does not affect GMP phosphorylation.***A*, Guanylyl cyclase activity assay. Purified recombinant RD3 was added to the retinal homogenates in the presence of 10 mM MgCl_2_ and 2 mM EGTA; *right inset*: Coomassie-stained 15% SDS-PAAG of the purified RD3; *left inset*: RetGC activity in the retina in the absence (●) and in the presence (○) of 5 μM RD3. *B,* GMP phosphorylation activity in *Rd3*^*+/+*^ wildtype (●,○), *rd3/rd3* (♦,◇), and *Rd3*^*+/+*^ line 362 inner retina (■, □) in the absence (●,♦, ■) or in the presence (○,◇, □) of 5 μM recombinant RD3; there was no detectable guanylate kinase activity in the RD3 preparation. The *p*-values are from Student’s unpaired/unequal variance *t* test. RD3, retinal degeneration 3 protein; RetGC, retinal membrane guanylyl cyclase.

The observed GMP phosphorylation rate in the retinas, regardless of the presence or absence of the endogenous RD3, exceeded that of the GTP to cGMP conversion by guanylyl cyclase. The RetGC activity in WT retinas averaged 0.9 nmol cGMP/min/retina, whereas in rd3/rd3 mice, it was reduced to 0.06 nmol/min/retina (Table 1). Adding RD3 to the WT retinal homogenates inhibited RetGC activity in a dose-dependent manner (IC_50_ = 0.05 ± 0.02 μM, *n* = 4), reducing it 4-fold at 5 μM (Fig. 5*A*). We did not detect any effect on the GMP phosphorylation rate in WT retinas or in *Rd3*^+/+^ retinas lacking photoreceptors using the same preparation of RD3 (Fig. 5*B*). In case the endogenous RD3 retinas masked the effect of added RD3, we also tested homozygous *rd3/rd3* (*Rd3*^*-/-*^) retinas (Fig. 5). To maximize the potential stimulating effect of RD3, the recombinant RD3 was added to the *rd3/rd3* retinal preparation that displayed the lowest GMP phosphorylation activity among the samples in the series (Fig. 4*C*, *asterisk*). Again, contrary to the hypothesis (20) (Fig. 1*B*), RD3 at the same concentration that strongly inhibited RetGC activity did not affect the rate of GMP phosphorylation in the mouse retinas lacking endogenous RD3 (Fig. 5*B*).

### Degeneration of the rd3 photoreceptors in the dark

**Figure 6:**
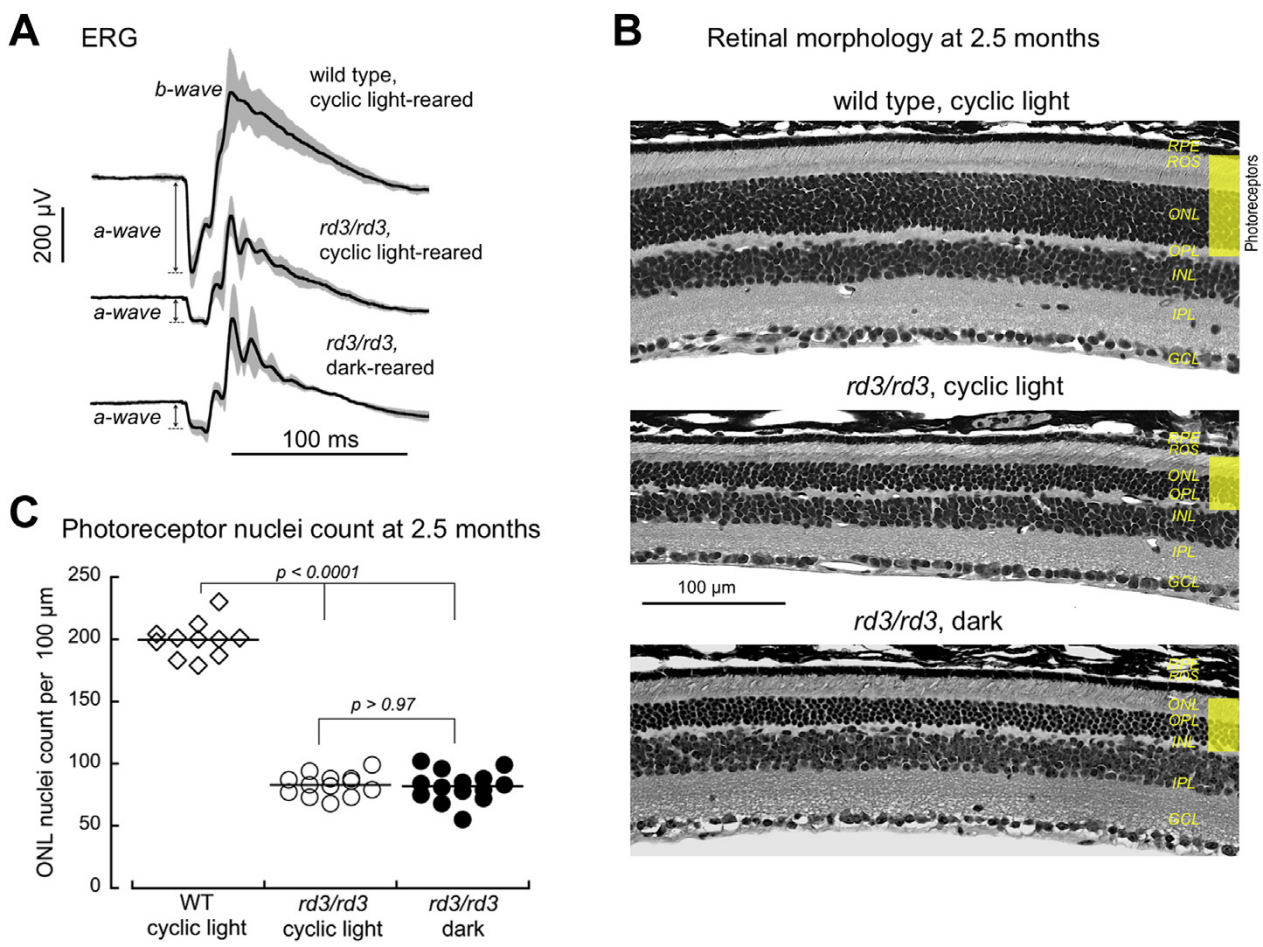
**Degeneration of the photoreceptor layer is unaffected in 2.5-month-old *rd3/rd3* mice reared in the dark.***A,* Prolonged dark adaptation does not improve ERG responses in *rd3/rd3* retinas. Dark-adapted rod-cone full-field ERG response to a bright flash (5.4 ×10^5^ photons/rod), averaged from different mice in each series (solid lines; *gray error bars—*standard deviation). The a-wave amplitude in *rd3/rd3* mice reared in the dark and under 12h dark/12h light cycle was equally suppressed (82 ± 14 μV, and 83 ± 24 μV, n = 5; *p* = 0.9992) when compared with wildtype (353 ± 62 μV, n = 6; *p* < 0.0001, ANOVA (F = 79, *p* < 0.0001)/Tukey’s HSD). *B,* Representative morphology of the retina, top to bottom: wildtype and *rd3/rd3* reared for 2.5 months under 12h dark/12h light cycle, and *rd3/rd3* mice reared for 2.5 months in the dark. C. Photoreceptor nuclei count in wildtype (◇) and *rd3/rd3* retinas (●,○) from mice reared under the cyclic light conditions (○,◇) or in the dark (●). All data points are from different mice; ANOVA (F = 382; *p* < 0.0001); the *p*-values in the graph are from the Tukey’s HSD *post hoc* test. ERG, electroretinography.

We further reasoned that if the depletion of GTP by the combined RetGC and PDE6 activities were an important factor contributing to the degeneration of RD3-deficient photoreceptors (20) (Fig. 1*B*), then rearing *rd3* mice in constant darkness from birth would have to reduce the pace of degeneration, by preventing activation of both PDE6 and RetGC. We hence randomized *rd3/rd3* mice from several litters at birth and housed them either under a regular 12-hour-dark/12-hour-light (30–50 lux) cycle or in complete darkness. Prolonged rearing in the dark did not improve electroretinography (ERG) in *rd3/rd3* mice compared with their littermates housed in the normal cyclic lighting conditions. The ERG a-wave (negative voltage deflection directly produced by hyperpolarizing photoreceptors) was equally reduced in both cases (Fig. 6*A*). There was no difference in the extent of photoreceptor degeneration, either. After 2.5 months of rearing in the dark, the reduction of photoreceptor nuclei count per 100 μm in *rd3/rd3* mice was exactly the same as in their littermates reared in the normal light cycle (82 ± 13, *n* = 13, *versus* 83 ± 9, *n* = 14, respectively; ANOVA/Tukey’s HSD *post hoc*, *p* > 0.97), a ∼59% loss compared with WT retinas (*p* < 0.0001) (Fig. 6).

## Discussion

Deficiency of RD3, a photoreceptor-specific protein located primarily in the inner segment (19, 24), has been linked to severe retinal disorders in animals and humans (1, 16, 32). The lack of RD3 reduces the overall content of RetGC in photoreceptors ((15, 17) and Table 1) by decreasing the efficiency of the cyclase delivery to the outer segment (2, 15). Photoreceptors in mice completely lacking RetGC degenerate much slower than in *rd3/rd3* mice, even though the latter still retain detectable RetGC activity and even demonstrate rudimentary photoresponses (17, 19). Hence, although the strong reduction of RetGC activity in the absence of RD3 can explain the impaired-from-birth function of the photoreceptors, it can only partially account for the rapid degeneration of the photoreceptors. Considering that (i) RD3 also strongly inhibits RetGC catalytic activity and its activation by Mg^2+^GCAPs (17, 18, 19) and (ii) the deletion of both GCAPs dramatically prolongs the survival of *rd3/rd3* photoreceptors (19, 24), it is most likely that the *rd3* and LCA12 photoreceptors degenerate because they cannot suppress RetGC activity in the inner segment in the absence of RD3 (Fig. 1*A*). However, Plana-Bonamaisó *et al.* proposed that it was specifically Mg^2+^GCAP2 that triggers the apoptotic process in the inner segment of *rd3* photoreceptors (24). We reasoned that if that were the case, GCAP2 deletion alone should rescue the RD3-deficient photoreceptors in a manner similarly to the dual deletion of both GCAP1 and GCAP2 (19, 24).

Although we observed a definite increase in the photoreceptor nuclei count after deleting GCAP2 (Fig. 2), that increase was rather minor. The photoreceptor layer was much better preserved in *rd3/rd3* mice lacking both GCAPs simultaneously and even better preserved in *rd3/rd3* mice lacking GCAP1 than in those lacking GCAP2 (Fig. 2). Both GCAP isoforms contribute to the activation of RetGC in mouse photoreceptors (25, 26, 33). In line with that, the changes in *rd3* retinal morphology observed after deleting GCAP1 or GCAP2 *versus* simultaneous deletion of both GCAPs (Fig. 2) suggest that RD3 is required to prevent activation of the cyclase by both GCAPs (Fig. 1*A*). These results do not support the hypothesis (24) that GCAP2 is the only or even the main factor in causing *rd3* degeneration.

Whereas the deregulation of RetGC (most likely in the inner segment (17, 18, 19)) emerges as the main culprit behind degeneration of the RD3-deficient photoreceptors (Fig. 1*A*), the events downstream of the aberrant RetGC activation remain unclear at this point. Hypothetically, cGMP in photoreceptor inner segment could prematurely activate cGMP-gated CNG channels produced in the endoplasmic reticulum and cause aberrant release of Ca^2+^ from the reticulum. Alternatively, the process could involve cGMP-dependent protein kinases. These and other possible scenarios would need to be evaluated in future in-depth studies.

Regarding the role of RD3 in protecting photoreceptors from degeneration (Fig. 1), a very interesting alternative hypothesis proposed by Wimberg *et al*. (20) stated that RD3 helps photoreceptors recycle GMP back to the pool of di- and thriphosphates, so in the absence of RD3, the guanylate kinase becomes inactive, and the GDP pool becomes depleted. Consequently, *rd3* and LCA12 photoreceptors would fail to restore GTP consumed by RetGC and PDE6 and degenerate. Using enzymatically coupled reactions to detect GMP conversion to GDP, the authors based that hypothesis on their observation that RD3 increased the activity in a commercial preparation of a porcine guanylate kinase and accelerated GMP phosphorylation 2-fold in a fraction of rod outer segments (20). We found this hypothesis (Fig. 1*B*) to be of particular interest, because it possibly suggested a different explanation for the protective effect of deleting GCAPs in *rd3* photoreceptors. Conceivably, deletion of GCAPs could, by reducing RetGC activity, decrease the rate of GTP consumption by the cyclase (and hence GMP production by PDE6). However, we also reasoned that to deplete GTP *via* RetGC activity (Fig. 1*B*), the rate of GMP phosphorylation would have to be much slower than the rate of GTP consumption by the cyclase or would at least have to become much slower in the absence of RD3. Our direct comparison of the rates of the GTP→cGMP conversion by RetGC and GMP phosphorylation shows that the two are indeed drastically different but in a manner directly opposite from that presumed by the hypothesis: GMP phosphorylation in the retina occurs at least 30-fold faster than the GTP consumption by RetGC (Fig. 4, Table 1).

Like PDE6, RetGC activity is exclusively present in photoreceptors (34, 35, 36, 37, 38), whereas GMP phosphorylation occurs in both photoreceptors and the inner retina (ref. (27) and Table 1). Hence, the comparison between the GMP phosphorylation and the cyclase in the entire retina would overestimate the rate of GMP phosphorylation over that of GTP consumption by RetGC. To better estimate the fraction of the guanylate kinase activity that belongs to photoreceptors, we subtracted GMP phosphorylating activity in the inner retina from that of the whole retina (Table 1). We utilized retinas devoid of photoreceptors to evaluate GMP phosphorylating activity in the inner retina (Fig. 4, Table 1), which amounted to ∼30% of the total retina activity, thus leaving the remaining ∼70% in the photoreceptor layer. Notably, that distribution was similar to the distribution of guanylate kinase activity in the dissected retinal layers from other mammalian species reported by Berger *et al*. Calculated from Figures 3 and 4S in reference (27), 71%–78% of guanylate kinase activity in rabbit and monkey retinas was associated with the photoreceptor inner segments and outer nuclear layer, and 22%–29% was associated with the outer plexiform layer. It is therefore possible that the bulk of GMP phosphorylation activity in the inner retina (Fig. 4, Table 1) is present in the dendrites of the secondary retinal neurons in the inner retina.

Even after we corrected the rate of GMP phosphorylation in photoreceptors by reducing it to 70% of the total activity in the retina, the rate of GMP phosphorylation attributable specifically to photoreceptors remains more than 20-fold higher than the rate of GTP consumption by RetGC (Fig. 4, Table 1) in WT mice. The balance between GTP consumption by RetGC and GMP phosphorylation shifts even further in *rd3* mice. Unable to properly accumulate RetGC in their outer segment, *rd3* photoreceptors reduce their RetGC activity to less than 10% the normal level (references (17, 19), Table 1). This creates another major obstacle for the hypothesis that GMP recycling is impaired in *rd3* photoreceptors (Fig. 1*B*), because GMP phosphorylation rate does not change in *rd3* photoreceptors (Figs. 4 and 5, Table 1), and it now exceeds that of GTP consumption by RetGC by a staggering 400-fold (Figs. 4 and 5, Table 1). Comparatively, a modest twofold stimulation of guanylate kinase by recombinant RD3 in retinal preparations reported in (20) would seem at best a rather minor addition to the already robust excess of GMP recycling activity over that of RetGC. Nonetheless, we were unable to detect even that small stimulation of GMP phosphorylation, contrary to the potent inhibitory effect of RD3 on RetGC activity (Fig. 5). The failure of the purified RD3 to stimulate GMP phosphorylation in WT retinas could be because of the presence of the endogenous retinal RD3 that already saturated the guanylate kinase activity in the assays. However, we excluded such a possibility as well. The GMP phosphorylation rates in WT and *rd3/rd3* retinas were virtually identical (Figs. 4*C* and 5*B*, Table 1), and RD3 did not stimulate GMP phosphorylation when added to the *rd3/rd3* retinal preparations lacking the endogenous RD3.

Last but not least, the phenotype of *rd3* photoreceptor degeneration remained unchanged in the dark (Fig. 6). This also undercuts the hypothesis that the lack of RD3 kills photoreceptors by preventing them from quickly restoring the guanosine diphosphate (and subsequently triphosphate) pool depleted by RetGC and PDE6 (20) (Fig. 1*B*). Both GTP conversion to cGMP by RetGC and cGMP hydrolysis by PDE6 are suppressed in the dark and accelerated in the light (6, 7, 13, 14). It is important to emphasize that the lighting conditions used in the experiment were fully sufficient to activate phototransduction during the light period of the cycle (39). Photoreceptors in *rd3/rd3* mice bred into C57B6 strain background used in this study degenerate in half between 2 and 3 months of age (19). If the rate of GTP depletion by RetGC were a major factor contributing to *rd3* photoreceptor degeneration, then rearing *rd3/rd3* mice in constant darkness since birth would be expected to significantly decelerate it. Contrary to such expectations from the Hypothesis 2 (Fig. 1*B*), the photoreceptors in *rd3/rd3* mice degenerated in the dark just as severely as did those of their littermates housed under normal-illumination conditions (Fig. 6).

To summarize, the results of our study support the hypothesis depicted in Figure 1*A* and rule out the alternative hypothesis depicted in Figure 1*B*. We conclude that (i) RD3 protects photoreceptors against degeneration by counteracting GCAP-dependent stimulation of the retinal membrane guanylyl cyclase; (ii) primarily GCAP1 and to a lesser extent GCAP2 both contribute to the triggering of photoreceptor degeneration in the absence of RD3; (iii) the rate of GMP phosphorylation in photoreceptors drastically exceeds the rate of GTP-to-GMP conversion *via* the RetGC/PDE6 pathway; and (iv) the role of RD3 in protecting photoreceptors from degeneration does not involve stimulation of GMP phosphorylation.

## Experimental procedures

### Animals

All experiments involving animals were conducted in accordance with the Public Health Service guidelines and approved by the Salus University Institutional Animal Care and Use Committee. C57B6J and *rd3/rd3* mouse strains were purchased from JAX Research/Jackson’s Laboratory; *GCAP1*^*-/-*^ (*Guca1a*^*-/-*^) and *GCAP2*^*-/-*^ (*Guca1b*^*-/-*^) mice harboring the respective gene disruption were developed as previously described (25, 26); *GCAP1,2*^*−/−*^ mice, in which the neighboring *Guca1a* and *Guca1b* genes were simultaneously deleted by substitution with a single PGK:Neo cassette (10) were kindly provided by Dr Jeannie Chen (University of Southern California). The *rd3* and transgenic strains used in this study were made congenic to the C57B6 genetic background by more than 10 generations of breeding to the C57B6 (17). Both males and females were used nonselectively in all experiments. Mice were housed using 12 h/12h dark/light (30–50 lux measured in different parts of the cages). Where indicated, they were reared in constant darkness from birth, except for several minutes per week exposure to the ambient light during changing the cages and a brief exposure to a dim red flashlight illumination when checking daily on the conditions of the animals.

### Genotyping

The presence of the *rd3*-specific C→T transition in exon 3 (1) was identified using a Sanger sequencing of the 0.6-kb *Rd3* gene fragment PCR-amplified from tail DNA samples by high-fidelity Phusion Flash DNA polymerase (Thermo Scientific) as previously described (19). *GCAP1*^*-/-*^, *GCAP2*^*-/-*^, and *GCAP1,2*^*−/−*^ were genotyped as previously described in detail (10, 25, 26).

### Retinal morphology

Mice were anesthetized with a lethal dose of Ketamine/Xylazine injection, perfused through the heart with phosphate buffered saline (PBS) and then with 2.5% glutaraldehyde/2.5% formaldehyde in PBS. The eyes were surgically removed and fixed overnight in 2.5% glutaraldehyde/2.5% formaldehyde/PBS solution (Electron Microscopy Sciences) at 4 °C. The fixed eyes were washed in PBS, soaked in PBS overnight, processed for paraffin embedding, sectioned (5 μm thickness), and stained with hematoxylin/eosin (AML Laboratories, Saint Augustine, FL). The retinal sections were photographed using an Olympus Magnafire camera mounted on an Olympus BX21 microscope. The photoreceptor nuclei in the outer nuclear layer of the retina were counted from 425 μm fragments of the retina between the optic nerve and the periphery, averaged from 2 to 3 fragments per retina, and the density of the nuclei per 100 μm length was averaged from multiple mice of each genotype as indicated in the figures.

### RD3 expression and purification

The recombinant RD3 was expressed from a Novagen pET11d vector in a BL21(DE3) Codon Plus *E. coli* strain (Stratagene/Agilent Technologies) induced by isopropyl–β–D-thiogalactopyranoside, extracted from the inclusion bodies, and purified by salt precipitation and dialysis as previously described in detail (18, 40, 41). The resultant purified RD3 was subjected to 15% SDS-PAAG electrophoresis and Coomassie staining. There was no detectable guanylate kinase activity in the recombinant RD3.

### GCAP expression and purification

Myristoylated bovine GCAP1 was expressed from pET11d vector in a BLR(DE3) *E. coli* strain (both from Novagen/Calbiochem) harboring a pBB131 plasmid coding for a yeast N-myristoyl transferase and purified by calcium precipitation, butyl–Sepharose and Sephacryl S–100 chromatography as described previously in detail (42, 43). The purity of GCAP1 preparations estimated by SDS gel electrophoresis was ≥90%.

### RetGC1 expression and guanylyl cyclase activity assays

Mouse retinas for RetGC activity measurements were excised from the dark-adapted 3.5-week-old mice under infrared illumination (Kodak number 11 infrared filters) using a dissecting microscope fitted with an Excalibur infrared goggles as described (17, 19), wrapped in aluminum foil, frozen in liquid N_2_, and stored at −70 °C before their use in the cyclase activity assays conducted under infrared illumination. The guanylyl cyclase activity was assayed as previously described in detail (44) with modification described in (17). In brief, the 25 μl assay mixture containing homogenate of 0.2 retina, 30 mM MOPS–KOH (pH 7.2), 60 mM KCl, 4 mM NaCl, 1 mM DTT, 2 mM EGTA, 10 mM Mg^2+^, 0.3 mM ATP, 4 mM cGMP, 1 mM GTP, 1 μCi of [α–^32^P]GTP, 0.1 μCi [8-^3^H]cGMP Perkin Elmer), 100 μM zaprinast and dipyridamole, 10 mM creatine phosphate and 0.5 unit of creatine phosphokinase (Sigma Aldrich) was incubated at 30 °C for 12 min. The reaction was stopped by heat-inactivation at 95° for 2 min, and a 5-μl aliquot from each reaction was chromatographed by TLC on fluorescently backed PEI cellulose plates (Merck) in 0.2 M LiCl. The spot containing the resultant [^32^P]cGMP and [8-^3^H]cGMP control radioactivity was excized from the plate and the [^32^P] and [^3^H] radioactivity eluted with 2 M LiCl were counted using liquid scintillation. Data fitting was performed using Synergy Kaleidagraph 4 software.

### 5’GMP phosphorylation assays

[8-^3^H]5’GMP substrate was prepared from 200 μCi [8-^3^H]cGMP (Perkin Elmer). The [8-^3^H]cGMP solution in 50% ethanol, 7 Ci/mmol, was dried to remove ethanol from the solution, dissolved in 120 μl of 50 mM HEPES KOH buffer (pH 7.5) containing 100 mM NaCl, 2 mM MgCl_2_, and 1 mM DTT and incubated for 30 min at 30 °C with 2 μg purified bovine PDE6 holoenzyme (a gift from Dr Nikolai Artemyev, University of Iowa). After the incubation, PDE6 was heat-inactivated for 2 min at 95°. At least 98% of [8-^3^H]cGMP was converted to [8-^3^H]GMP when tested by TLC on PEI cellulose plates in 0.2 M LiCl (Fig. 3). Mouse retinas excised from 3.5-weeks-old mice were homogenized in 100 mM Tris/HCl, pH 8.0 containing 20 mM MgCl_2_, 120 mM KCl, proteases inhibitors, 20 mM creatine phosphate, and 1 unit of creatine phosphokinase and were used for the phosphorylation assay immediately or after being aliquoted, frozen in liquid N_2_, and stored at −70 °C. The 20-μl assay mixture containing 0.015 retinas per reaction in 50 mM Tris/HCl, pH 8.0, 10 mM MgCl_2_, 60 mM KCl, 1 mM GMP, 2 mM ATP, ∼0.3 μCi of [^3^H]GMP, proteases inhibitors, 10 mM creatine phosphate, and 0.5 unit of creatine phosphokinase (Sigma Aldrich) was incubated at 30 °C for 15 to 20 min. The reaction was stopped by adding of 5 ul of 5% trifluoroacetic acid, neutralized by adding 5 μl of 0.4 M Na_2_CO_3_, and 5-μl aliquots were chromatographed on PEI cellulose TLC plates developed in 0.5 M LiCl. The spots containing ^3^H-GDP and ^3^H-GTP were excised from the PEI cellulose plate, and the [^3^H] radioactivity eluted with 0.5 ml 2 M LiCl was counted using liquid scintillation in 10 ml of scintillation cocktail. Data fitting was performed using Synergy Kaleidagraph 4 software.

### Dark-adapted electroretinography

ERG was performed in the dark as previously described in detail (30, 31) using a Phoenix Research Laboratories Ganzfeld ERG2 instrument. The mice reared under cyclic lighting conditions or in the dark were housed overnight in complete darkness before the ERG recordings. The pupils were dilated by applying 1% Tropicamide and 2.5% Phenylephrine ophthalmic eye drops under dim red safelight illumination, and the mice were dark-adapted for another 10 min. Full-field ERG was recorded in mice anesthetized by inhalation of 1.7 to 1.9% Isoflurane (VEDCO) delivered using a Kent Scientific SmnoSuite setup. Bright (5.4 × 10^5^ photoisomerizations/rod) 1-ms 505-nm light pulses were delivered through infrared camera-guided corneal electrode/LED light source. The a-wave amplitude was measured 7 ms after the flash.

### Statistics

ANOVA and *post-hoc* Tukey’s honestly significance difference (HSD) test and nonpaired/unequal variance Student’s *t* test were performed using Synergy Kaleidagraph 4 and StatPages on-line resource. Data normality and homogeneity of variances before applying the ANOVA/*post hoc* testing were verified using the respective Kolmogorov-Smirnov and Levene’s tests.

## Data availability

The data referred to in this manuscript are contained within the manuscript. Unprocessed data can be available from the corresponding author (adizhoor@salus.edu) upon reasonable request.

## Conflict of interest

The authors declare that they have no conflicts of interest with the contents of this article.
